# Clear Aligners in the Growing Patient: A Systematic Review

**DOI:** 10.3390/children11040385

**Published:** 2024-03-23

**Authors:** Alessio Danilo Inchingolo, Gianna Dipalma, Irene Ferrara, Fabio Viapiano, Anna Netti, Anna Maria Ciocia, Antonio Mancini, Giuseppina Malcangi, Andrea Palermo, Angelo Michele Inchingolo, Francesco Inchingolo

**Affiliations:** 1 Department of Interdisciplinary Medicine, School of Medicine, University of Bari “Aldo Moro”, 70124 Bari, Italy; ad.inchingolo@libero.it (A.D.I.); giannadipalma@tiscali.it (G.D.); irene.ferrara@uniba.it (I.F.); Fabio.viapiano@uniba.it (F.V.); anna.netti@uniba.it (A.N.); annamaria.ciocia@uniba.it (A.M.C.); antonio.mancini@uniba.it (A.M.); a.inchingolo2@studenti.uniba.it (A.M.I.); francesco.inchingolo@uniba.it (F.I.); 2 College of Medicine and Dentistry, Birmingham B4 6BN, UK; andrea.palermo2004@libero.it

**Keywords:** clear aligner treatment, mucogingival modifications, aesthetic, growing patients, arch form development, malocclusion, transverse discrepancies, class II malocclusion, gingival morphology, acceptability of aligners

## Abstract

Mixed dentition represents a critical phase in the oral development of pediatric patients, characterized by the simultaneous presence of primary and permanent teeth. This article proposes a comprehensive systematic review of the application of aligners as an innovative methodology in managing mixed dentition. The primary objective is to explore the efficacy, safety, and acceptability of this emerging orthodontic technology in the evolving age group. This systematic review focuses on randomized controlled trials, cohorts, and observational studies investigating the use of aligners in patients with mixed dentition. Clinical, radiographic, and psychosocial parameters will be considered to assess the overall impact of aligner therapy in this critical phase of dental development. An in-depth analysis of such data aims to provide a comprehensive overview of the potential of this technology in pediatric orthodontics. Expected outcomes may contribute to outlining practical guidelines and targeted therapeutic strategies for orthodontists involved in managing mixed dentition. Furthermore, this article aims to identify gaps in the current research and suggest future directions for studies exploring the use of transparent aligners in patients with mixed dentition, thereby contributing to the ongoing evolution of evidence-based orthodontic practices.

## 1. Introduction

Orthodontic aligners have revolutionized the field of orthodontics, providing an aesthetically pleasing and comfortable solution for the treatment of malocclusions. In recent years, there has been a growing interest in the use of aligners in growing patients, including those with mixed and primary dentition [1,2].

Mixed dentition represents a pivotal moment in the oral development of pediatric patients, characterized by the coexistence of primary and permanent teeth [3,4].

This phase, which generally occurs between the ages of 6 and 12, is crucial for early diagnosis and orthodontic intervention to prevent or correct occlusal and functional anomalies [5,6].

In this period, orthodontic professionals are often called upon to manage the unique challenges posed by an ever-evolving oral environment.

The evolution of orthodontic technologies has introduced the use of clear aligners, known for their discretion and comfort, as a potential solution for orthodontic management in this age group [7,8].

In recent decades, orthodontics has witnessed significant advancements in techniques and materials, among which clear aligners stand out. These devices represent a revolution in the field of orthodontics, offering an aesthetic and less invasive solution compared to traditional fixed appliances [9,10]. The popularity of aligners is growing, especially among young patients seeking a less invasive and more aesthetically pleasing option [11,12]. However, the application of aligners in growing patients remains relatively unexplored, with studies focused predominantly on adolescents and adults. The realm of orthodontics has been profoundly transformed by the advent of clear aligner technology, offering a blend of aesthetic appeal and comfort for patients undergoing treatment for various malocclusions [13,14]. Early orthodontic intervention, particularly with aligners, is an area ripe with potential yet fraught with challenge [15,16]. The judicious application of aligners in young patients can navigate the complex waters of conditions such as posterior crossbite, Class III dentoskeletal discrepancies, and excessive overjet, which may benefit from timely and interceptive treatment strategies [17,18].

The efficacy, safety, and acceptability of aligners in this particular stage of dental development require thorough analysis. Recent research has begun to investigate the applicability of aligners in patients with mixed dentition, evaluating various aspects such as dental movement control, patient experience, and clinical outcomes [11,19]. However, the literature on this topic is still fragmented and requires a systematic review to synthesize the available evidence [9,20].

Early orthodontic treatment continues to be a debated topic in the world of orthodontics, primarily due to uncertainties regarding its benefits and long-term stability [21]. However, the possibility of correcting or improving malocclusions at an early stage, albeit challenging, remains very appealing [22,23]. Pediatricians are generally the first healthcare professionals to refer young patients for orthodontic consultation, serving as important links between families and orthodontic specialists. Since many malocclusions have a genetic basis, the primary goal of early treatment is to create a more favorable environment for jaw and dental arch growth [24]. The benefits of early treatment include the ability to leverage the skeletal adaptability of growing children and the improvement to dental and facial aesthetics, thus enhancing quality of life and psychosocial well-being [25]. Furthermore, early treatment can reduce the duration and complexity of future orthodontic treatments, facilitating the subsequent treatment phase. The optimal timing of treatment and the choice of appropriate therapeutic strategies represent the main challenges, along with patient compliance [26,27].

The clinical focus will be the effect of aligners on dental development, evaluating parameters such as efficacy in correcting malocclusions, the impact on periodontal health, and the incidence of dental caries [28,29]. In addition, radiographic aspects, such as monitoring root position and jaw bones, are considered crucial for a thorough analysis of the impact of aligners [30,31].

From a psychosocial perspective, the acceptance of orthodontic treatment and its impact on the quality of life of pediatric patients are of no less significance. Aligners, due to their discretion and comfort, could play a significant role in enhancing the orthodontic experience of young patients [32]. This systematic review aims to bridge this gap by critically examining existing studies on the use of aligners in mixed dentition and growing patients. Through the analysis of randomized controlled trials, cohorts, and observational studies, this work aims to provide a comprehensive overview of the use of aligners in this delicate phase of orthodontic development. Additionally, the article seeks to identify gaps in current research and suggest future directions for studies, thereby contributing to the evolution of evidence-based orthodontic practices.

## 2. Materials and Methods

### 2.1. Search Processing

The International Prospective Register of Systematic Review Registry (ID: 520127) and PRISMA principles were followed in this systematic review. Using an English-language criterion, we searched PubMed, Cochrane Library, Scopus, and Web of Science for pertinent papers published between 1 January 2014 and 1 January 2024. We used a combination of search terms specific to the goal of the investigation. As a result, the Boolean terms used were ((children) AND (aligners) AND (orthodontic)).

### 2.2. Inclusion Criteria

Reviewers carried out a comprehensive analysis, rating all qualifying trials according to the subsequent inclusion standards: (1) randomized control trials (RCTs) or randomized controlled clinical trials (RCCTs); (2) human participant studies; (3) full-text articles available for free; and (4) English-language publications. On the other hand, the following exclusion criteria were determined: (1) systematic or literature reviews; (2) editorials; (3) case reports and case series; (4) in vitro articles; (5) animal-related studies; and (6) articles not released in English. The characteristics outlined according to the Participant, Intervention, Comparison, Outcome, Time (PICOT) framework were as follows: (1) pediatric patients with malocclusion issues in primary and mixed dentition stages; (2) orthodontic treatment with clear aligners or other orthodontic interventions; (3) comparisons between different types of orthodontic treatments or between clear aligners and traditional appliances; (4) various outcomes including treatment efficacy, treatment duration, patient satisfaction, dental and skeletal changes, and safety; (5) studies published between 1 January 2014 and 1 January 2024. These strict guidelines were used in the article selection process to make sure the studies chosen for the systematic review fulfilled the requirements for relevance and quality.

## 3. Results

**Figure 1:**
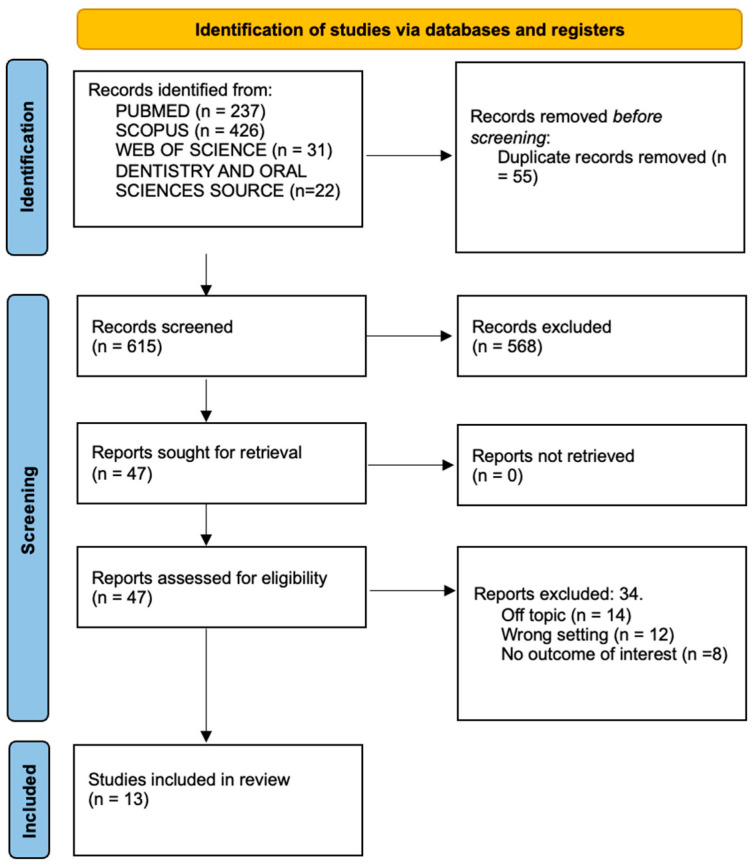
PRISMA flowchart of the inclusion process. PRISMA Checklist is available in Supplementary Materials.

A total of 716 papers were found after a thorough search of several databases, including PubMed (237), Scopus (426), Dentistry & Oral Sciences Source (22), and Web of Science (31). A total of 661 unique items were left after duplicates (55) were eliminated. After a thorough review of abstracts and titles, 615 publications were excluded. The remaining 46 publications were then successfully retrieved by the authors, who also evaluated their suitability. Thirty-one publications were eliminated during this process since they were not relevant to the subject. In the end, the evaluation contained 15 studies for qualitative analysis (Figure 1 and Table 1).

## 4. Discussion

Orthodontic interventions in the primary and mixed dentition phases, particularly with the use of clear aligners, have become a focal point of research and discussion. This comprehensive exploration delves into various aspects, including the benefits and challenges of early orthodontic treatment with aligners, the effectiveness of orthodontic expansion techniques, strategies for addressing Class II malocclusions, the impact of aligners on gingival and periodontal modifications, and the overall effectiveness, safety, and acceptability of aligners in primary and mixed dentition.

### 4.1. Early Benefits and Challenges of Early Orthodontic Treatment with Aligners

Early orthodontic treatment remains a subject of debate, yet the potential to rectify malocclusions in their initial stages is undeniably appealing [46]. Conditions such as posterior crossbite, Class III dentoskeletal issues, impacted teeth, and excessive overjet can all benefit from interceptive treatment [47,48]. The primary aim of early treatment is to establish a conducive growth environment, enhancing aesthetics and minimizing future complexities [49].

Early orthodontic treatment effectively addresses interceptive orthodontic issues in growing patients, generally aligning with the planned ClinCheck^®^ predictions [50,51].

However, treatment success is significantly influenced by complexity, with more challenging corrections necessitating additional aligners [52,53].

### 4.2. Orthodontic Expansion

Orthodontic interventions for palatal expansion are crucial in addressing transverse discrepancies and optimizing dental harmony in growing patients [54].

Recent studies have explored the efficacy and predictability of palatal expansion using clear aligners in children with mixed dentition, offering valuable insights for orthodontic practitioners [55,56].

Kim’s retrospective study [44] analyzed 164 patient records, providing a nuanced understanding of arch expansion predictability with the Invisalign First^®^ system. Their research emphasized variations between the maxillary and mandibular arches, highlighting the importance of meticulous treatment planning. Factors such as the predicted expansion per aligner and the strategic placement of attachments significantly influenced treatment outcomes [57,58].

Attachment placement on upper first permanent molars emerged as a critical determinant for achieving efficient tooth movement [44].

Lione’s prospective study [39] investigated orthodontic treatment with Invisalign First System^®^ aligners during the early mixed dentition phase. Their research, involving 23 patients, demonstrated significant transverse dimensional changes, underscoring the effectiveness of Invisalign First System^®^ in expanding the maxillary arch during this critical developmental stage [39].

Lu’s prospective cohort study [40] compared the treatment effects of Invisalign First System with a traditional Rapid Maxillary Expander (RME) in mixed dentition cases. The study revealed that the Invisalign group exhibited substantial dental and dentoalveolar changes, positioning it as an effective option for mild to moderate maxillary transverse discrepancies [59].

However, their research underscored a preference for RME in severe discrepancies due to its superior expansion capabilities [60].

Levrini’s preliminary study [35] explored the efficacy of Invisalign^®^ First clear aligners in achieving palatal expansion during mixed dentition. Patients, treated for an average of 8 months, demonstrated significant changes in various measurements, showcasing the potential of clear aligners in increasing arch width. The study acknowledged the comfort and aesthetic benefits of Invisalign^®^ First compared to traditional fixed appliances, suggesting further research to validate these promising results [61,62].

This preliminary exploration encourages a reevaluation of treatment modalities for mild crowding or limited transverse maxillary deficiencies in growing patients [35].

### 4.3. Class II Malocclusion

Class II malocclusion, characterized by mandibular hypoplasia or retraction, poses a common orthodontic challenge [63].

Various functional appliances have been utilized to address sagittal dysregulation and minimize the need for future orthognathic surgery.

Recent advancements by Align Technology™ have introduced the Invisalign mandibular advancement (MA), offering an invisible alternative with superior aesthetics, comfort, and precision compared to traditional appliances [64].

This section explores the effectiveness of MA in treating Class II malocclusion, comparing it with traditional functional appliances.

Sadek’s retrospective study [37] focused on evaluating the efficacy of Invisalign^®^ mandibular advancement in treating Class II malocclusion with mandibular retrusion. Analyzing 67 cases, this study showcased significant improvements in skeletal vertical relationships and dental relationships.

Yue’s retrospective study [42] investigated the impact of Invisalign mandibular advancement (MA) and Twin-Block (TB) treatments on upper airway morphology and hyoid bone position in skeletal Class II malocclusion [65].

The study, involving 32 children, emphasized the importance of a three-dimensional (3D) evaluation using cone-beam computed tomography (CBCT) data. Both treatments showed significant increases in oropharynx and hypopharynx volumes, with potential advantages of MA in relieving hypopharynx airway obstruction [66].

The findings underscore the significance of individualized treatment approaches in addressing airway concerns associated with skeletal Class II malocclusion [42].

Wu et al. [43] conducted a retrospective study comparing the skeletal and dentoalveolar effects of various appliances, including the Invisalign mandibular advancement (MA), Vanbeek Activator, Herbst, and Twin-Block, in children with Skeletal Class II malocclusion [67].

The Vanbeek Activator showed more skeletal improvement for deep overjets, while Twin-Block and MA were particularly effective in bone effects for correcting Class II molar occlusion [68].

Zhang et al.’s study [45] compared the effects of clear functional aligners and Twin-Block on temporomandibular joint (TMJ) parameters in adolescent Class II division 1 malocclusion, providing valuable insights into their potential in stimulating condylar growth.

Clear functional aligners showed a more significant increase in condylar height, while Twin-Block exhibited a greater increase in the length of the mandibular rami and anterior and posterior diameters of the condyle [45,69].

### 4.4. Impact of Aligners on Gingival and Periodontal Modifications

Orthodontic interventions have traditionally been aimed at achieving dental alignment, but contemporary approaches also consider their effects on gingival and periodontal health.

Recent studies explore the nuanced impact of clear aligner systems, particularly the Invisalign First System, on gingival morphology modifications. Additionally, a comparative analysis is presented, highlighting the influence of orthodontic appliances, including removable aligners and fixed options, on periodontal health.

Lione et al. [36] conducted a prospective evaluation to assess the influence of the Invisalign First System on gingival morphology during Phase I orthodontic treatment in growing patients. The study, involving 18 subjects with early mixed dentition, observed significant reductions in the gingival margin height (GMH) for various teeth and increased deciduous canine inclination (DCI). These findings highlight the aesthetic impact of Invisalign First treatment on smiles and suggest potential positive effects on gingival aesthetics, although the study acknowledges its limitations [36,70].

Abbate’s comparative analysis [33] investigated the impact of orthodontic appliances on periodontal health, specifically comparing fixed orthodontic appliances to removable aligners. This study emphasized the drawbacks associated with fixed appliances, including increased plaque accumulation and subsequent periodontal issues. In contrast, removable aligners, such as Invisalign^®^, were noted for facilitating better oral hygiene maintenance. The clinical trial involving 50 teenagers reveals significantly lower plaque and bleeding scores in the Invisalign^®^ group, suggesting superior periodontal health and enhanced compliance with oral hygiene practices compared to fixed appliances [33].

These studies contribute to understanding the evolving paradigm of orthodontic care, emphasizing the intricate interplay between orthodontic treatments and gingival or periodontal health. Clear aligners, such as the Invisalign First System, not only serve as tools for achieving dental aesthetics but also potentially contribute to improved periodontal well-being compared to traditional fixed appliances [71].

### 4.5. Effectiveness, Safety, and Acceptability of Aligners

Clear aligners have revolutionized orthodontics, providing an aesthetically pleasing solution that has gained popularity, particularly among younger patients [1,7].

Despite their widespread use, the application of aligners in primary and mixed dentition remains an underexplored territory.

This section aims to scrutinize studies evaluating the effectiveness, safety, and acceptability of aligners during this critical developmental phase [72,73].

Merino da Silva’s randomized clinical trial [41] compared the efficacy of clear aligners to traditional fixed appliances in resolving maxillary incisor irregularities during mixed dentition. Despite predictability rates ranging from 48.7% to 61.1%, the study found a comparable efficacy between the two groups [41].

Chou et al. [34] investigated the efficacy and efficiency of clear aligner treatment (CAT) versus fixed appliance treatment (FAT) in adolescents with moderate to severe malocclusions. CAT exhibited shorter treatment durations and fewer scheduled visits compared to FAT, suggesting its efficiency in treating malocclusions in adolescents [34].

Dianiskova’s study [38] explored patient and parental satisfaction following orthodontic treatment with clear aligners (CAs) and elastodontic appliances (EAs) during childhood/adolescence. Overall satisfaction was reported with both treatments, with distinctions emerging regarding comfort and social impact. These insights provide valuable considerations for aligner treatments during mixed dentition [38].

In summary, these studies contribute to our understanding of the effectiveness, safety, and acceptability of aligners in the intricate context of primary and mixed dentition, providing clinicians and families with evidence-based considerations in orthodontic decision making.

## 5. Limitations and Future Directions

In this systematic review of orthodontic treatment with aligners in pediatric patients, several inherent limitations are apparent. Heterogeneity among the included studies poses a significant challenge, as they exhibit wide variations in design, patient characteristics, treatment protocols, and assessed outcomes. This heterogeneity complicates direct comparisons and impedes the attainment of definitive conclusions. Limited sample sizes in certain studies may restrict the generalizability of our findings and statistical power, rendering it difficult to discern significant differences. Furthermore, the duration of follow-up varies across studies, with some offering only short-term data, while others lack long-term data on treatment stability and efficacy. Moreover, the absence of high-quality randomized controlled trials directly comparing aligners with traditional fixed appliances restricts our ability to ascertain the relative effectiveness and long-term outcomes of treatment within the purview of this review. While this systematic review provides a comprehensive examination of orthodontic treatment with clear aligners in pediatric patients, encompassing various aspects including benefits, challenges, expansion techniques, management of Class II malocclusions, and impact on gingival and periodontal health, it is crucial to acknowledge and address these inherent limitations. Future research directions may entail larger randomized controlled trials to enhance the understanding of movement predictability in early mixed dentition treated with clear aligners. Additionally, further investigations are warranted to refine treatment guidelines and compare patient perceptions across different approaches.

## 6. Conclusions

The presented studies provide valuable insights into the benefits and challenges associated with early orthodontic treatment utilizing aligners, particularly in addressing various malocclusion issues during primary and mixed dentition phases. The findings underscore the potential of interceptive orthodontic interventions to rectify malocclusions in their initial stages, thereby minimizing future complexities and enhancing aesthetics. Furthermore, studies exploring orthodontic expansion, Class II malocclusions, and the impact of aligners on gingival and periodontal health provide further evidence of the efficacy and safety of aligner treatments across different orthodontic conditions. Clear aligners emerge as not only effective tools for achieving dental aesthetics but also potentially contribute to improved periodontal well-being compared to traditional fixed appliances. Moreover, research evaluating the effectiveness, safety, and acceptability of aligners during primary and mixed dentition phases indicates comparable efficacies to traditional fixed appliances, with potential advantages in terms of treatment duration and patient comfort. These findings underscore the importance of evidence-based orthodontic decision making and highlight the evolving paradigm of orthodontic care, with clear aligners playing a significant role in meeting the needs of growing patients. However, further research, particularly randomized controlled trials with larger sample sizes, is recommended to enhance understanding and optimize treatment outcomes in early orthodontic intervention with aligners.

## Figures and Tables

**Table 1:** Descriptive summary of item selection.

Author (Year)	Study Design	Number of Patients	Average Age	Type of Aligners	Malocclusion	Treatment Time	Treatment Performed	Outcomes Aligner Patients
Abbate et al. (2015) [33]	RCT	50	10–18 years old	Invisalign^®^ aligners	Not specified	12 months	Group 1: Invisalign^®^ aligners (n = 25) Group 2: Traditional fixed brackets (n = 25)	Compliance with oral hygiene: better Plaque index: lower Gingival inflammation: lower
Chou et al. (2020) [34]	Case control study	72 (47 clear aligner treatment and 25 fixed appliance treatment).	13 y.o.	Invisalign^®^ Aligners	Class I and II malocclusions with moderate to severe discrepancy	Clear aligner treatment group: 24 ± 6 months, fixed appliance treatment group: 27 ± 5 months	Clear aligner treatment or fixed appliance treatment chosen by patients, including the use of interarch elastics and/or Class II–correction appliances, extractions	Treatment time: shorter Incisor proclination: lower
Levrini et al. (2021) [35]	Case control study	20 patients (12 F, 8 M)	8.9 years (range: 6.9 to 11.2 years)	Invisalign^®^ First	Maxillary arch width, arch perimeter, arch depth, molar inclination, alveolar expansion	8 months	Maxillary expansion using Invisalign^®^ First clear aligners	Intercanine and deciduous molar widths: increased.
Lione et al. (2022) [36]	Case control study	18 subjects (10 F, 8 M)	9.4 years (±1.2)	Invisalign First System^®^	Early mixed dentition with mild to moderate crowding	7.8 months (±2.4)	Sequential anterior crowding, and alignment with Invisalign aligners	Gingival inflammation: lower Intercanine and deciduous expansion of arches, correction of molar widths: increased.
Sadek et al. (2022) [37]	Case control study	15	11,5 years old	Invisalign^®^ Aligners with mandibular advancement feature	Class II division 1 malocclusion characterized by retrusion of the mandible	17.73 months	Non-extraction treatment modality with Invisalign^®^ mandibular advancement	SNB angle: increased ANB angle: increased, Upper incisor–palatal plane angle: decreased Overjet: decreased Upper lip protrusion: improved.
Dianiskova et al. (2023) [38]	Cross-sectional Case–Control Study	56	10 y.o.	Elastodontic Appliances, Clear Aligners	Various types of malocclusions, including crowding, excessive overjet, distal bites, and dentoalveolar discrepancies	Treatment duration varied	Orthodontic treatment during mixed dentition with elastodontic appliances and clear aligners.	School and social life: improved. Grinding: reduced Breathing: improved.
Lione et al. (2023) [39]	Case control study	23	9.4 years old	Invisalign First System^®^	Posterior transverse discrepancy between maxillary and mandibular arches up to 6 mm, mixed dentition	8.1 months	Nonextraction treatment with Invisalign First System^®^ clear aligners, employing sequential upper arch expansion	Deciduous molar and canine widths: increased Rotating upper first molars: effective
Lu et al. (2023) [40]	Cohort study	51 patients (Invisalign First ^®^ group 17; RME group 17; Natural growth group: 17)	Not specified	Invisalign First ^®^ system	Maxillary transverse deficiency in mixed dentition	6 months	First group: Treatment with Invisalign First System RME group: Treatment with acrylic splint rapid maxillary expander (RME)	Invisalign First is suitable for mild to moderate transversal deficiencies, while RME is more efficient for severe cases.
Merino da Silva et al. (2023) [41]	RCT	32 patients enrolled, 27 completed treatment	9.33 years for the clear aligners group; 9.65 years for the fixed appliances group	Clear aligners and partial (2 × 4) fixed appliance	Maxillary incisor position irregularities in the mixed dentition	8 months	Correction of maxillary incisor irregularities using clear aligners or fixed 2 × 4 appliances	Maxillary incisor crowding: same effectiveness with both appliances.
Yue et al. (2023) [42]	Case control study	32 children (15 M and 17 F)	10.2 ± 0.84 years	Invisalign mandibular advancement (MA) and Twin-Block (TB) appliances	Skeletal Class II malocclusion characterized by mandibular deficiency	MA group—11.45 ± 1.1 months; TB group—12.11 ± 1.3 months	Evaluation of the changes in the upper airway and hyoid bone position before and after treatment with MA and TB	Enlargement of the oropharynx and hypopharynx airway segments in both appliances MA was found to be more effective than TB in dilating the upper airway hypopharynx obstruction site.
Wu et al. (2023) [43]	Case control study	63 patients in mixed or early permanent dentition	Not specified	Mandibular advancement (MA), Vanbeek Activator, Herbst, and Twin-Block	Skeletal Class II malocclusion	Not specified	Comparison of skeletal and dentoalveolar effects of MA, Vanbeek Activator, Herbst, and Twin-Block in children with Skeletal Class II Malocclusion	Advanced the mandible, improved facial profile, and corrected skeletal Class II MA primarily corrected Class II through dentoalveolar effects with incisor inclination control.
Kim et al. (2024) [44]	Case control study	90	8.42 years old	Invisalign First^®^	Arch expansion in children with early mixed dentition	Not specified	Arch expansion using the Invisalign First^®^ system	Expansion effectiveness: better on molar deciduous than first permanent molar.
Zhang et al. (2024) [45]	Case control study	49	10.89 years old	Invisalign	Class II division 1 malocclusion with mandibular retrusion	Not explicitly stated	Functional orthodontic treatment with Twin-Block or clear functional aligners	Stimulated condylar growth and sagittal/vertical mandibular development. Twin-Block induced a more significant increase in mandibular ramus length No substantial articular fossa remodeling was noted. Condylar position shifted, with retrodisplaced condyle relocating to the middle.

## Data Availability

Not applicable.

## Supplementary Materials

The following are available online at https://www.mdpi.com/article/10.3390/children11040385/s1, PRISMA 2020 Checklist.

## Abbreviations

AC	Anterior Crossbite
ANB	A point-Nasion-B point
CAT	Clear Aligner Treatment
CBCT	Cone-Beam Computed Tomography
CRE	Cast Radiograph Evaluation
DCI	Deciduous Canine Inclination
EA	Elastodontic Appliances
F	Female
FAT	Fixed Appliance Treatment
GMH	Gingival Margin Height
M	Males
MA	Mandibular Advancement
PROMs	Patient-Reported Outcome Measures
RME	Rapid Maxillary Expander
SN	Sella-Nasion
TB	Twin-Block
TMJ	Temporomandibular Joint
Y.O.	Years Old
